# Crystal structure of a bioactive sesquiterpene isolated from *Artemisia reticulata*

**DOI:** 10.1107/S2056989016003236

**Published:** 2016-03-04

**Authors:** A. K. Bauri, Sabine Foro, Nhu Quynh Nguyen Do

**Affiliations:** a Bioorganic Division, Bhabha Atomic Research Centre, Trombay, Mumbai 85, India; b Clemens Schöpf-Institut für Organische, Chemie und Biochemie, Technische Universität Darmstadt, Petersenstrasse 22, D-64287 Darmstadt, Germany; c Accident & Emergency Department, Franco, Vietnamese Hospital, 7-Nguyen, Luong Bang Street, HoChiMinh City, Vietnam

**Keywords:** crystal structure, sesquiterpene, isolation, *Artemisia reticulata*, anti­proliferative property

## Abstract

The title compound, C_15_H_24_O_2_ {systematic name: 1-[6-hy­droxy-7-(propan-2-yl)-4-methyl­idene-2,3,3a,4,5,6,7,7a-octa­hydro-1*H*-inden-1-yl]ethanone} was iso­la­ted from *A. reticulata* by column chromatography over silica gel by gradient solvent elution. The mol­ecule comprises a bi­cyclo­[4.3.0]nonane ring bearing acet­oxy, hy­droxy and isopropyl substituents, and an exocyclic double bond on the cyclo­hexane ring. In the bicyclic skeleton, the cyclo­hexane ring adopts a chair conformation ring and the cyclo­pentane ring is in an envelope conformation. In the crystal, mol­ecules are linked by O—H⋯O hydrogen bonds, forming chains along [010]. These chains are cross-linked by C—H⋯O hydrogen bonds.

## Abstract 2

The sesquiterpene mol­ecule has been isolated from Indian herb *A. reticulata* by column chromatography over silica gel with a mixture of binary solvent ethyl acetate and hexane by gradient elution. It was recrystallized at room temperature by slow evaporation to afford suitable crystal for X-ray diffraction study. Anti­proliferative bioassay of this mol­ecule has been conducted against human ovarian cancer cell line A 2780.

## Chemical context   

The title compound is a natural product, which has been isolated from the Indian herb *A. reticulata* by column chroma­tography over silica gel. *A. reticulata* (family: *Asteraceae*) is a traditional herb which has many applications in folklore medicine for conventional therapy against several diseases such as malaria (Klayman *et al.*, 1984 ▸; Malagon *et al.*, 1997 ▸; Newton & White, 1999 ▸), cancer (Efferth *et al.*, 2001 ▸; Lai *et al.*, 1995 ▸), cardiovascular (Guantai *et al.*, 1999 ▸), vasodilatory (Walker, 1996 ▸), hepatitis (Aniya *et al.*, 2000 ▸) and diabetes (Iriadam *et al.*, 2006 ▸). It is found as a constituent in many ayurvedic or herbal drug preparations such as *forkolin* and *Afsanteen* in Indian traditional medicinal systems (Nadkarni, 1954 ▸; Satyavati *et al.*, 1987 ▸; Subramoniam *et al.*, 1996 ▸; Drury, 1978 ▸). The *Artemisia* species are a rich source of bioactive sesquiterpenenoids (Klayman *et al.*, 1984 ▸) such as artemisinin, artemisin *etc*. Artimisinin and artemisin are secondary metabolites isolated from herbs of the species *A. annua* (Klayman, 1985 ▸) belonging to the sesquiterpene class. The title mol­ecule possesses anti­plasmodial activity and it is now under clinical trial for the treatment of malaria. Our group are currently searching for artemisin, artemisinin or their analogues from other varieties of *Artemisia* species and as part of these studies, the structure of the title compound is now reported.

## Structural commentary   

**Figure 1:**
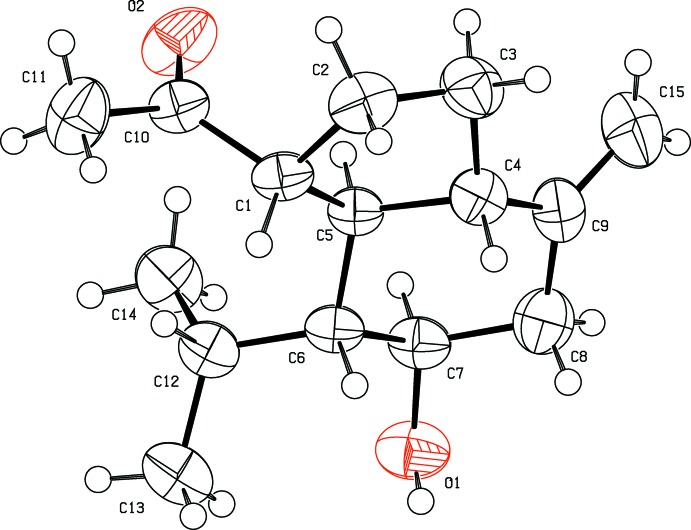
The mol­ecular structure of the title compound, showing 50% probability displacement ellipsoids for non-H atoms.

The mol­ecular structure of the title compound is shown in Fig. 1 ▸. The compound comprises fused cyclo­hexane and cyclo­pentane rings. It has been substanti­ated by a positive LB test (Liebermann Burchard Test), which indicates that it belongs to the sesquiterpene class. The compound is soluble in chloro­form but has poor solubility in methanol. 
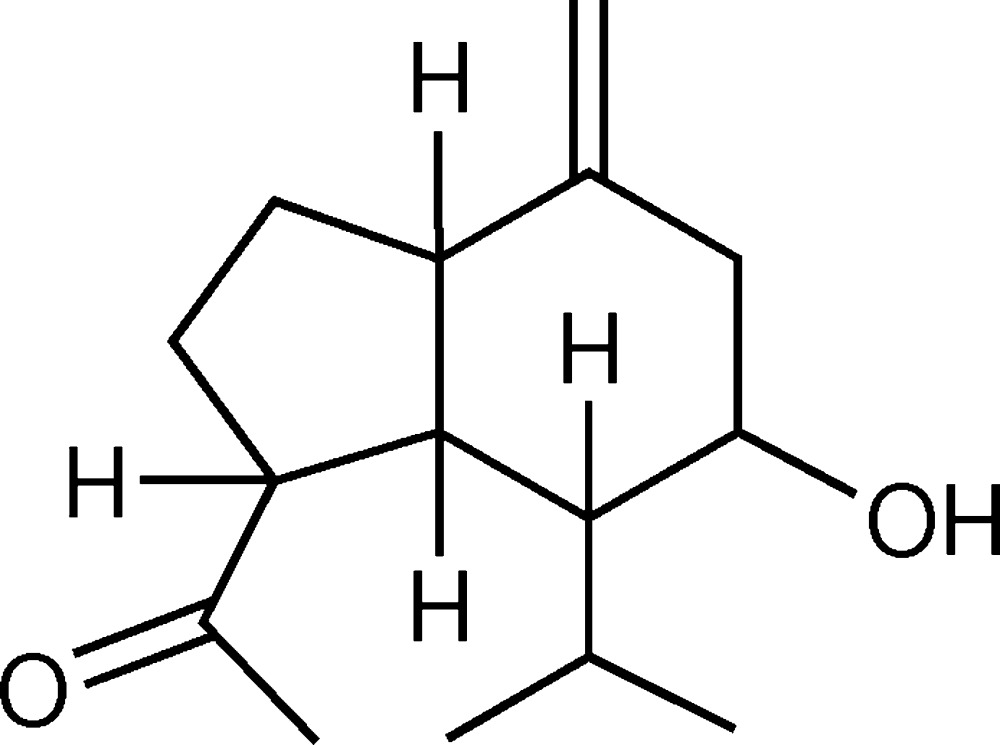


The bicyclic skeleton contains one acetyl group at atom C1 of the cyclo­pentane ring, one isopropyl group and one hydroxyl group located at atoms C6 and C7 in the cyclo­hexane ring. An exocyclic olefinic double bond is located between atoms C9 and C15 and attached to the cyclo­hexane ring. The torsion angles C3—C4—C5—C6 and C9—C4—C5—C1 of −169.2 (3) and −170.9 (3)°, respectively, describe the geometry at the junction of the two rings. The C7—C6—C5 and C9—C4—C5 angles are 107.3 (2) and 109.2 (3)°, respectively.

## Supra­molecular features   

**Figure 2:**
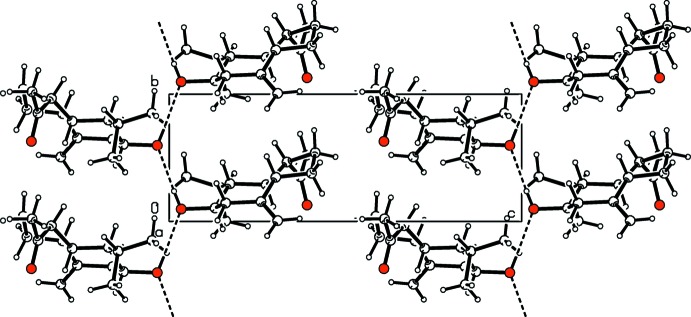
Part of the crystal structure of the title compound, with hydrogen bonds shown as dashed lines.

In the crystal, mol­ecules are linked by O—H⋯O hydrogen bonds, forming chains along [010] (Table 1 ▸ and Fig. 2 ▸). These chains are cross-linked by weak C—H⋯O hydrogen bonds.

## Database survey   

A search of Cambridge Structural Database (CSD, Version 5.36, last update May 2015; Groom & Allen, 2015 ▸) found only one mol­ecule, Pulioplopane A (15-hy­droxy-10 (14)-oplopen-4-one; Triana *et al.*, 2005 ▸) that has a similar structural skeleton to the title sesquitertene although it is is unrelated in a biochemical sense.

## Synthesis and crystallization   

The title sesquiterpene was isolated as colourless solid from the methanol extract of *A. reticulata* by chromatography over silica gel with a mixture of ethyl acetate and hexane with a gradient elution followed by preparative thin layer chromatography. Crystals were obtained after recrystallization three times from ethyl acetate:hexane (1:4) at room temperature by the slow evaporation method. Bioassay of this mol­ecule has been conducted against human ovarian cancer cell line A 2780 and revealed that it possessed significant anti­proliferative activity (unpublished results).

## Refinement   

Crystal data, data collection and structure refinement details are summarized in Table 2 ▸. H atoms were placed in calculated positions with C—H = 0.93–0.98 Å and O—H = 0.82 Å and refined in a riding-motion approximation with *U*_iso_(U) = 1.2*U*_eq_(C,O). No Friedel pairs were collected therefore the absolute configuration could not be determined from the X-ray data and the assignment is arbitrary.

## Supplementary Material

Crystal structure: contains datablock(s) global, I. DOI: 10.1107/S2056989016003236/lh5803sup1.cif

Structure factors: contains datablock(s) I. DOI: 10.1107/S2056989016003236/lh5803Isup2.hkl

Supporting information file. DOI: 10.1107/S2056989016003236/lh5803Isup3.cml

CCDC reference: 1455684

Additional supporting information:  crystallographic information; 3D view; checkCIF report

## Figures and Tables

**Table 1:** Hydrogen-bond geometry (Å, °)

*D*—H⋯*A*	*D*—H	H⋯*A*	*D*⋯*A*	*D*—H⋯*A*
O1—H1*O*⋯O1^i^	0.82	2.11	2.927 (4)	175
C11—H11*C*⋯O2^ii^	0.96	2.53	3.430 (6)	157

Symmetry codes: (i) 
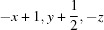
; (ii) 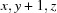.

**Table 2:** Experimental details

Crystal data	
Chemical formula	C_15_H_24_O_2_
*M*_r_	236.34
Crystal system, space group	Monoclinic, *P*2_1_
Temperature (K)	299
*a*, *b*, *c* (Å)	8.849 (4), 5.336 (1), 14.994 (5)
β (°)	99.21 (2)
*V* (Å^3^)	698.9 (4)
*Z*	2
Radiation type	Cu *K*α
μ (mm^−1^)	0.56
Crystal size (mm)	0.50 × 0.18 × 0.15

Data collection	
Diffractometer	Enraf–Nonius CAD-4
No. of measured, independent and observed [*I* > 2σ(*I*)] reflections	1916, 1392, 1260
*R*_int_	0.052
(sin θ/λ)_max_ (Å^−1^)	0.597

Refinement	
*R*[*F* ^2^ > 2σ(*F* ^2^)], *wR*(*F* ^2^), *S*	0.060, 0.164, 1.10
No. of reflections	1392
No. of parameters	154
No. of restraints	1
H-atom treatment	H-atom parameters constrained
Δρ_max_, Δρ_min_ (e Å^−3^)	0.46, −0.22

Computer programs: *CAD-4-PC* (Enraf–Nonius, 1993 ▸), *REDU4* (Stoe & Cie, 1987 ▸), *SHELXS97* and *SHELXL97* (Sheldrick, 2008 ▸) and *PLATON* (Spek, 2009 ▸).

## supplementary crystallographic information

### Crystal data

**Table:**

C_15_H_24_O_2_	*F*(000) = 260
*M**_r_* = 236.34	*D*_x_ = 1.123 Mg m^−^^3^
Monoclinic, *P*2_1_	Cu *K*α radiation, λ = 1.54180 Å
Hall symbol: P 2yb	Cell parameters from 25 reflections
*a* = 8.849 (4) Å	θ = 5.5–27.1°
*b* = 5.336 (1) Å	µ = 0.56 mm^−^^1^
*c* = 14.994 (5) Å	*T* = 299 K
β = 99.21 (2)°	Rod, colourless
*V* = 698.9 (4) Å^3^	0.50 × 0.18 × 0.15 mm
*Z* = 2	

### Data collection

**Table:**

Enraf–Nonius CAD-4 diffractometer	*R*_int_ = 0.052
Radiation source: fine-focus sealed tube	θ_max_ = 67.0°, θ_min_ = 3.0°
Graphite monochromator	*h* = −10→3
ω/2θ scans	*k* = 0→6
1916 measured reflections	*l* = −17→17
1392 independent reflections	3 standard reflections every 120 min
1260 reflections with *I* > 2σ(*I*)	intensity decay: 1.0%

### Refinement

**Table:**

Refinement on *F*^2^	Primary atom site location: structure-invariant direct methods
Least-squares matrix: full	Secondary atom site location: difference Fourier map
*R*[*F*^2^ > 2σ(*F*^2^)] = 0.060	Hydrogen site location: inferred from neighbouring sites
*wR*(*F*^2^) = 0.164	H-atom parameters constrained
*S* = 1.10	*w* = 1/[σ^2^(*F*_o_^2^) + (0.1147*P*)^2^ + 0.0812*P*] where *P* = (*F*_o_^2^ + 2*F*_c_^2^)/3
1392 reflections	(Δ/σ)_max_ < 0.001
154 parameters	Δρ_max_ = 0.46 e Å^−^^3^
1 restraint	Δρ_min_ = −0.22 e Å^−^^3^

### Special details

**Table:**

Geometry. All e.s.d.'s (except the e.s.d. in the dihedral angle between two l.s. planes) are estimated using the full covariance matrix. The cell e.s.d.'s are taken into account individually in the estimation of e.s.d.'s in distances, angles and torsion angles; correlations between e.s.d.'s in cell parameters are only used when they are defined by crystal symmetry. An approximate (isotropic) treatment of cell e.s.d.'s is used for estimating e.s.d.'s involving l.s. planes.
Refinement. Refinement of *F*^2^ against ALL reflections. The weighted *R*-factor *wR* and goodness of fit *S* are based on *F*^2^, conventional *R*-factors *R* are based on *F*, with *F* set to zero for negative *F*^2^. The threshold expression of *F*^2^ &gt; σ(*F*^2^) is used only for calculating *R*-factors(gt) *etc*. and is not relevant to the choice of reflections for refinement. *R*-factors based on *F*^2^ are statistically about twice as large as those based on *F*, and *R*- factors based on ALL data will be even larger.

### Fractional atomic coordinates and isotropic or equivalent isotropic displacement parameters (Å^2^)

**Table:**

	*x*	*y*	*z*	*U*_iso_*/*U*_eq_	
O1	0.5467 (3)	0.0974 (5)	0.03391 (14)	0.0580 (7)	
H1O	0.5259	0.2380	0.0135	0.070*	
O2	0.9664 (3)	0.1293 (5)	0.3910 (2)	0.0680 (8)	
C1	0.8020 (3)	0.4666 (6)	0.33401 (19)	0.0401 (7)	
H1	0.8184	0.6168	0.2992	0.048*	
C2	0.7177 (4)	0.5402 (8)	0.4141 (2)	0.0539 (9)	
H2A	0.7821	0.5069	0.4715	0.065*	
H2B	0.6912	0.7167	0.4112	0.065*	
C3	0.5742 (4)	0.3792 (9)	0.4035 (2)	0.0568 (9)	
H3A	0.5943	0.2188	0.4335	0.068*	
H3B	0.4923	0.4633	0.4277	0.068*	
C4	0.5350 (3)	0.3473 (7)	0.3022 (2)	0.0446 (7)	
H4	0.5020	0.5109	0.2765	0.053*	
C5	0.6918 (3)	0.2887 (5)	0.27514 (17)	0.0355 (6)	
H5	0.7191	0.1174	0.2952	0.043*	
C6	0.6880 (3)	0.2967 (5)	0.17229 (17)	0.0370 (7)	
H6	0.6501	0.4629	0.1518	0.044*	
C7	0.5672 (4)	0.1059 (6)	0.13003 (18)	0.0439 (7)	
H7	0.6039	−0.0600	0.1517	0.053*	
C8	0.4103 (4)	0.1446 (9)	0.1602 (2)	0.0590 (10)	
H8A	0.3654	0.2991	0.1341	0.071*	
H8B	0.3429	0.0081	0.1371	0.071*	
C9	0.4206 (4)	0.1563 (8)	0.2604 (2)	0.0508 (8)	
C10	0.9549 (4)	0.3515 (7)	0.3722 (2)	0.0431 (7)	
C11	1.0900 (4)	0.5203 (9)	0.3893 (3)	0.0671 (11)	
H11A	1.1664	0.4637	0.3552	0.081*	
H11B	1.1313	0.5185	0.4526	0.081*	
H11C	1.0594	0.6878	0.3713	0.081*	
C12	0.8473 (4)	0.2670 (6)	0.1447 (2)	0.0447 (8)	
H12	0.9160	0.3774	0.1847	0.054*	
C13	0.8571 (5)	0.3509 (9)	0.0487 (2)	0.0604 (10)	
H13A	0.8294	0.5246	0.0418	0.072*	
H13B	0.7882	0.2524	0.0067	0.072*	
H13C	0.9598	0.3286	0.0371	0.072*	
C14	0.9125 (5)	0.0040 (8)	0.1601 (3)	0.0613 (10)	
H14A	0.8454	−0.1135	0.1251	0.074*	
H14B	0.9217	−0.0377	0.2230	0.074*	
H14C	1.0116	−0.0027	0.1419	0.074*	
C15	0.3418 (5)	0.0067 (11)	0.3076 (3)	0.0708 (12)	
H15A	0.2759	−0.1134	0.2780	0.085*	
H15B	0.3528	0.0225	0.3701	0.085*	

### Atomic displacement parameters (Å^2^)

**Table:**

	*U*^11^	*U*^22^	*U*^33^	*U*^12^	*U*^13^	*U*^23^
O1	0.0792 (15)	0.0505 (15)	0.0419 (11)	−0.0110 (14)	0.0024 (10)	−0.0077 (11)
O2	0.0730 (17)	0.0337 (14)	0.0893 (19)	0.0019 (14)	−0.0110 (13)	0.0124 (14)
C1	0.0543 (16)	0.0251 (14)	0.0392 (13)	−0.0004 (13)	0.0022 (12)	0.0020 (12)
C2	0.067 (2)	0.045 (2)	0.0479 (16)	0.0059 (17)	0.0046 (14)	−0.0101 (16)
C3	0.0614 (19)	0.063 (2)	0.0489 (17)	0.0040 (18)	0.0176 (14)	−0.0047 (18)
C4	0.0501 (16)	0.0376 (17)	0.0472 (16)	0.0042 (15)	0.0112 (12)	−0.0022 (14)
C5	0.0451 (14)	0.0226 (14)	0.0386 (14)	−0.0002 (12)	0.0059 (11)	0.0015 (12)
C6	0.0513 (16)	0.0226 (14)	0.0369 (13)	0.0002 (13)	0.0064 (11)	0.0013 (12)
C7	0.0570 (17)	0.0330 (17)	0.0410 (14)	−0.0066 (15)	0.0052 (13)	−0.0001 (14)
C8	0.0527 (18)	0.062 (3)	0.0603 (19)	−0.010 (2)	0.0022 (14)	−0.004 (2)
C9	0.0423 (15)	0.049 (2)	0.0626 (18)	−0.0012 (16)	0.0135 (13)	−0.0002 (17)
C10	0.0516 (17)	0.0324 (16)	0.0438 (15)	−0.0007 (14)	0.0026 (12)	−0.0008 (14)
C11	0.0541 (19)	0.047 (2)	0.095 (3)	−0.0055 (18)	−0.0023 (18)	−0.002 (2)
C12	0.0551 (17)	0.0307 (16)	0.0492 (17)	−0.0035 (15)	0.0108 (13)	−0.0007 (14)
C13	0.078 (2)	0.052 (2)	0.0559 (19)	−0.004 (2)	0.0248 (17)	−0.0006 (18)
C14	0.070 (2)	0.042 (2)	0.077 (2)	0.0144 (19)	0.0264 (18)	0.0068 (19)
C15	0.060 (2)	0.076 (3)	0.080 (2)	−0.014 (2)	0.0229 (18)	−0.004 (2)

### Geometric parameters (Å, º)

**Table:**

O1—C7	1.424 (3)	C7—C8	1.542 (5)
O1—H1O	0.8200	C7—H7	0.9800
O2—C10	1.219 (5)	C8—C9	1.493 (5)
C1—C10	1.513 (4)	C8—H8A	0.9700
C1—C5	1.535 (4)	C8—H8B	0.9700
C1—C2	1.562 (5)	C9—C15	1.335 (6)
C1—H1	0.9800	C10—C11	1.486 (5)
C2—C3	1.521 (6)	C11—H11A	0.9600
C2—H2A	0.9700	C11—H11B	0.9600
C2—H2B	0.9700	C11—H11C	0.9600
C3—C4	1.513 (4)	C12—C14	1.521 (5)
C3—H3A	0.9700	C12—C13	1.523 (5)
C3—H3B	0.9700	C12—H12	0.9800
C4—C9	1.501 (5)	C13—H13A	0.9600
C4—C5	1.539 (4)	C13—H13B	0.9600
C4—H4	0.9800	C13—H13C	0.9600
C5—C6	1.538 (3)	C14—H14A	0.9600
C5—H5	0.9800	C14—H14B	0.9600
C6—C7	1.537 (4)	C14—H14C	0.9600
C6—C12	1.540 (4)	C15—H15A	0.9300
C6—H6	0.9800	C15—H15B	0.9300
			
C7—O1—H1O	109.5	C6—C7—H7	106.8
C10—C1—C5	114.5 (3)	C8—C7—H7	106.8
C10—C1—C2	108.6 (2)	C9—C8—C7	112.9 (3)
C5—C1—C2	105.1 (2)	C9—C8—H8A	109.0
C10—C1—H1	109.5	C7—C8—H8A	109.0
C5—C1—H1	109.5	C9—C8—H8B	109.0
C2—C1—H1	109.5	C7—C8—H8B	109.0
C3—C2—C1	105.8 (3)	H8A—C8—H8B	107.8
C3—C2—H2A	110.6	C15—C9—C8	123.8 (4)
C1—C2—H2A	110.6	C15—C9—C4	124.0 (4)
C3—C2—H2B	110.6	C8—C9—C4	112.2 (3)
C1—C2—H2B	110.6	O2—C10—C11	121.0 (3)
H2A—C2—H2B	108.7	O2—C10—C1	121.3 (3)
C4—C3—C2	102.8 (3)	C11—C10—C1	117.7 (3)
C4—C3—H3A	111.2	C10—C11—H11A	109.5
C2—C3—H3A	111.2	C10—C11—H11B	109.5
C4—C3—H3B	111.2	H11A—C11—H11B	109.5
C2—C3—H3B	111.2	C10—C11—H11C	109.5
H3A—C3—H3B	109.1	H11A—C11—H11C	109.5
C9—C4—C3	121.8 (3)	H11B—C11—H11C	109.5
C9—C4—C5	109.2 (3)	C14—C12—C13	109.7 (3)
C3—C4—C5	102.5 (2)	C14—C12—C6	113.3 (3)
C9—C4—H4	107.5	C13—C12—C6	114.6 (3)
C3—C4—H4	107.5	C14—C12—H12	106.2
C5—C4—H4	107.5	C13—C12—H12	106.2
C1—C5—C6	118.0 (2)	C6—C12—H12	106.2
C1—C5—C4	103.8 (2)	C12—C13—H13A	109.5
C6—C5—C4	112.5 (2)	C12—C13—H13B	109.5
C1—C5—H5	107.3	H13A—C13—H13B	109.5
C6—C5—H5	107.3	C12—C13—H13C	109.5
C4—C5—H5	107.3	H13A—C13—H13C	109.5
C7—C6—C5	107.3 (2)	H13B—C13—H13C	109.5
C7—C6—C12	115.3 (3)	C12—C14—H14A	109.5
C5—C6—C12	113.1 (2)	C12—C14—H14B	109.5
C7—C6—H6	106.9	H14A—C14—H14B	109.5
C5—C6—H6	106.9	C12—C14—H14C	109.5
C12—C6—H6	106.9	H14A—C14—H14C	109.5
O1—C7—C6	113.9 (2)	H14B—C14—H14C	109.5
O1—C7—C8	109.0 (2)	C9—C15—H15A	120.0
C6—C7—C8	112.9 (3)	C9—C15—H15B	120.0
O1—C7—H7	106.8	H15A—C15—H15B	120.0
			
C10—C1—C2—C3	−116.4 (3)	C5—C6—C7—C8	52.9 (3)
C5—C1—C2—C3	6.5 (4)	C12—C6—C7—C8	179.9 (3)
C1—C2—C3—C4	−31.5 (4)	O1—C7—C8—C9	−179.9 (3)
C2—C3—C4—C9	166.7 (3)	C6—C7—C8—C9	−52.2 (4)
C2—C3—C4—C5	44.5 (4)	C7—C8—C9—C15	−124.2 (4)
C10—C1—C5—C6	−95.1 (3)	C7—C8—C9—C4	53.1 (4)
C2—C1—C5—C6	145.9 (3)	C3—C4—C9—C15	2.1 (6)
C10—C1—C5—C4	139.6 (3)	C5—C4—C9—C15	121.1 (4)
C2—C1—C5—C4	20.5 (3)	C3—C4—C9—C8	−175.3 (3)
C9—C4—C5—C1	−170.9 (3)	C5—C4—C9—C8	−56.3 (4)
C3—C4—C5—C1	−40.5 (3)	C5—C1—C10—O2	−31.7 (5)
C9—C4—C5—C6	60.4 (3)	C2—C1—C10—O2	85.3 (4)
C3—C4—C5—C6	−169.2 (3)	C5—C1—C10—C11	150.5 (3)
C1—C5—C6—C7	−179.0 (3)	C2—C1—C10—C11	−92.5 (4)
C4—C5—C6—C7	−58.0 (3)	C7—C6—C12—C14	−53.3 (4)
C1—C5—C6—C12	52.8 (3)	C5—C6—C12—C14	70.7 (4)
C4—C5—C6—C12	173.7 (3)	C7—C6—C12—C13	73.6 (4)
C5—C6—C7—O1	178.0 (2)	C5—C6—C12—C13	−162.4 (3)
C12—C6—C7—O1	−55.0 (3)		

### Hydrogen-bond geometry (Å, º)

**Table:**

*D*—H···*A*	*D*—H	H···*A*	*D*···*A*	*D*—H···*A*
O1—H1*O*···O1^i^	0.82	2.11	2.927 (4)	175
C11—H11*C*···O2^ii^	0.96	2.53	3.430 (6)	157

Symmetry codes: (i) −*x*+1, *y*+1/2, −*z*; (ii) *x*, *y*+1, *z*.
